# Osteoid Osteoma with a Multicentric Nidus: Interstitial Laser Ablation under MRI Guidance

**DOI:** 10.1155/2013/254825

**Published:** 2013-05-30

**Authors:** David Kaul, Oriane Bonhomme, Phillip Schwabe, Bernhard Gebauer, Florian Streitparth

**Affiliations:** ^1^Department of Radiology, Charité—University Medicine Berlin, Augustenburger Platz 1, 13353 Berlin, Germany; ^2^Center for Musculoskeletal Surgery, Charité—University Medicine Berlin, Augustenburger Platz 1, 13353 Berlin, Germany

## Abstract

Osteoid osteoma (OO) is a common benign tumor of the bone and is typically treated by thermal ablation with computed tomography (CT) guidance. Only a few cases of multicentric OO have been described. We here report the case of an 11-year-old boy with multicentric OO of the right femur treated with laser ablation under open high-field MRI guidance. The steps of the interventional MRI procedure are described, discussing the benefits and disadvantages of MRI versus CT guidance especially with regard to younger patients.

## 1. Introduction

Osteoid osteoma (OO) is a benign neoplasm of the bone that makes up about 12% of all benign bone tumors [1]. The tumor typically affects young male adults and presents with severe night pain that responds well to salicylates. Most OOs occur in the long bones especially the femur and the tibia. The tumor usually consists of a single nidus of highly vascularized osteoid tissue surrounded by sclerotic bone. In some cases, an OO may present with more than one nidus; in this case, the tumor is referred to as multicentric. To date, only 24 cases of multicentric OO in a single bone have been described [2]. 

The main treatment options for OO include en bloc resection and curettage of the nidus. Since the extent of surgical resection is often disproportional to the actual tumor size, [3] thermal ablation of OO has become more popular since computed tomography- (CT-) guided radiofrequency ablation (RFA) was first described in 1992 [4–6]. Other recently described treatment options include CT-guided thermal laser ablation [7]. Since CT guidance has the obvious disadvantage of radiation exposure, which is especially problematic in younger patients, and restricted imaging options, magnetic resonance imaging (MRI) guidance has recently been used for minimally invasive interventions. Open MRI has played a major role in this development since it provides better patient access than conventional MRI [8]. We here report a case of multicentric osteoid osteoma treated by laser ablation in an open MRI system.

## 2. Case Report

**Figure 1:**
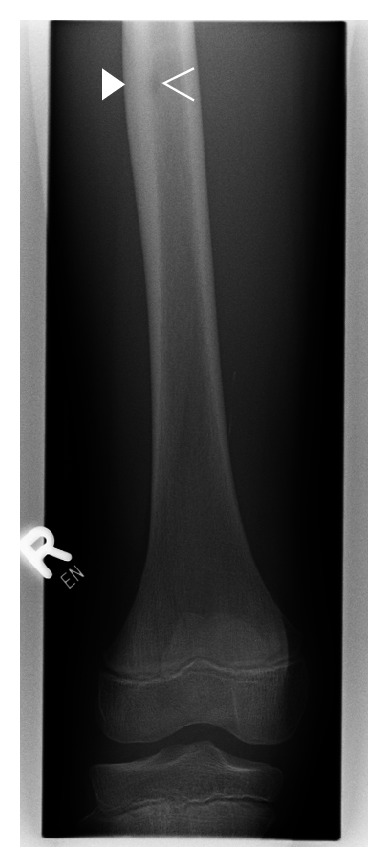
X-ray of the knee and the distal femur shows an osteoid osteoma-like lesion with typical cortical reaction (solid arrowhead) and a suspected central lucency (open arrowhead).

**Figure 2:**
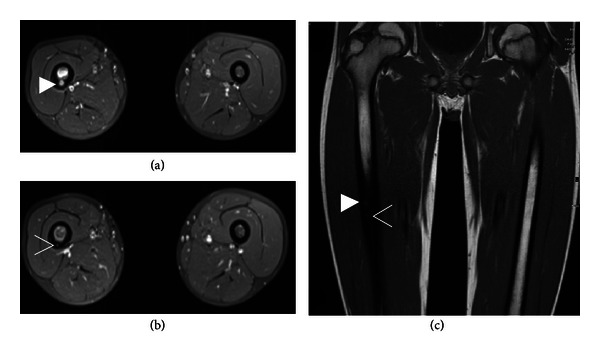
Contrast-enhanced MRI reveals a multicentric osteoid osteoma with two nidi in the femur diaphysis with a larger nidus of 6 × 5 mm (solid arrowhead in (a) and (c)) and a smaller 2 × 2 mm nidus inferiorly (open arrowhead in (b) and (c)). Note the thickened cortical bone and the bone marrow edema of the right femoral diaphysis. (a) and (b) contrast-enhanced T1 fs MRI; (c) T1 TSE MRI.

An 11-year-old boy was referred to our department with a more than one-year history of local pain in the right leg which worsened at night. He explained that the pain had started after a minor accident in which he had fallen on his knee. A radiograph of the knee had not revealed any abnormality (not shown). The patient did not tolerate salicylates and thus had been treated with acetaminophen, which had only given him minor relief. A second X-ray, which included not only the knee but also the distal femur, identified thickened cortical bone and a suspected nidus in the diaphysis of the femur (Figure 1). An MRI scan showed the typical appearance of OO of the right femur diaphysis with a nidus measuring 6 × 5 mm. A second nidus, measuring 2 × 2 mm, was identified, establishing the diagnosis of multicentric OO. The superoinferior distance between the two lesions was 8.5 mm (Figure 2). We offered to treat the patient using laser ablation under open MRI guidance and monitoring [9]. The patient and his parents were informed of possible complications and treatment alternatives by an orthopedic surgeon and an interventional radiologist. Informed consent was obtained. The intervention was performed as described elsewhere [9]. In brief, the open interventional MRI system (1.0 T Panorama HFO, Philips Healthcare, Best, The Netherlands) includes the MRI scanner, a workstation with full scanner control, an in-room monitor, an MR-compatible Bluetooth mouse for operator-controlled sequence initiation, and a single-loop surface coil.

**Figure 3:**
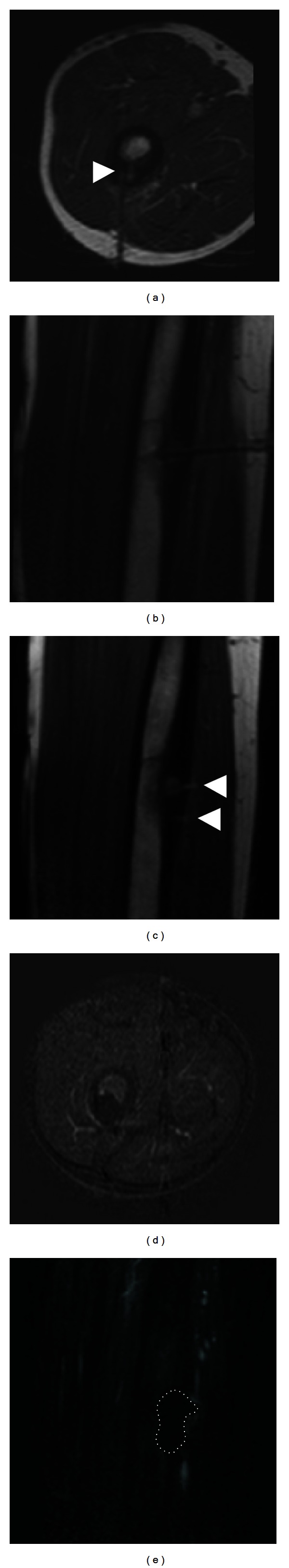
A fast dynamic T1w TSE sequence (image acquisition 2 s) was used for verifying proper needle/laser placement in the target lesion ((a) and (b)). Note the needle artifact with projection of the needle tip onto the nidus (solid arrowhead in (a)). Note the additional benefit of multiplanar navigation in a parasagittal plane (b). Both drilling holes can be seen in this parasagittal plane after the procedure (solid arrowheads in (c)). Subtraction images of unenhanced T1-w TSE and contrast-enhanced T1-w TSE were generated to confirm complete ablation by the absence of nidal contrast enhancement ((d) and dotted areas in (e)).

Before the procedure, prophylactic antibiosis was administered intravenously (cefuroxim-ratiopharm 1.5 g, Ulm, Germany). The whole intervention was performed under general anesthesia. To reduce postoperative pain, subcutaneous and periperiosteal local anesthesia (Xylonest 1%, AstraZeneca, Wedel, Germany) was administered. A fast T1-weighted turbo spin echo (T1w TSE) sequence (TE/TR 5.7/200 ms, TF 7, fa 90, scan duration 2 s) was used for instrument guidance during the intervention (Figure 3). An MRI-compatible bone biopsy drill (11 G, Somatex, Teltow, Germany) was used to create an access to the two nidi in freehand technique. 18-G coaxial needles (Somatex, Teltow, Germany) were used to enter the nidi through the biopsy channels with real-time MRI guidance. A 400 um bare laser fiber (Frank Optic Products, Berlin, Germany) was introduced through the needle. After 5 mm retraction of the needle its position was confirmed again, and laser treatment with an Nd:YAG laser (1064 nm, Fibertom Medilas, Dornier MedTech, Wessling, Germany) was started, applying a continuous energy flow and an effective output of 2.5 watts. The higher nidus was treated with an energy of 1200 J, applied over 8 min, while the lower nidus received 750 J over 5 min. Temperature tissue effects were monitored in near real-time using a thermosensitive T1-weighted fat-saturated gradient echo (T1w fs GRE) sequence, enabling image update every 4 s. Postinterventionally, subtraction images of unenhanced and contrast-enhanced T1w TSE sequences showed an appropriate ablation zone with a typical loss of nidal contrast enhancement, confirming therapeutic success (Figure 3). 

The two nidi were successfully localized, targeted, and treated under MRI guidance. The laser effect was depicted online as previously described [9]. No complications occurred. The patient was symptom-free after treatment. After the procedure, the patient was monitored for 2 hours in the recovery room. He was discharged from the hospital 24 h after treatment. 

The whole procedure was performed in 97 min. A total of 6 pulse sequences were acquired, including a survey, T2w SPIR TSE for planning, PDw TSE for interactive drilling and needle/laser positioning, T1w GRE for MR thermometry, and finally pre- and postcontrast T1w TSE for computing subtraction images.

**Figure 4:**
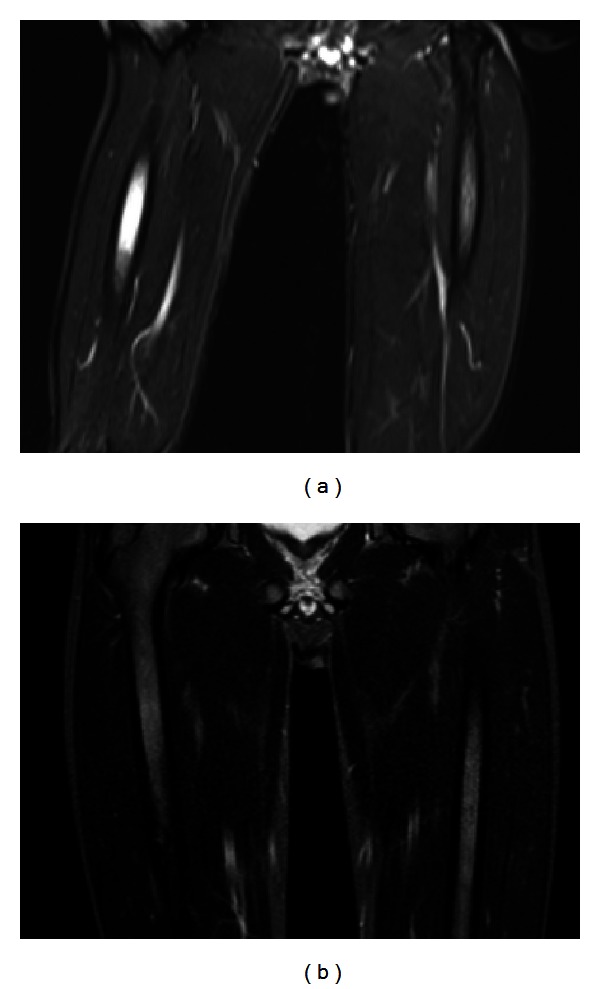
At 6-month followup, the patient remains asymptomatic. The edema of the bone marrow before the procedure (a) has completely diminished (b). T2w STIR sequences in coronal slice orientations.

At 6-month followup, the patient remains asymptomatic, without the need for oral anti-inflammatory agents. MRI showed complete resolution of femoral bone marrow edema, consistent with the relief of symptoms (Figure 4). 

## 3. Discussion

Traditional surgical treatment methods include open “en bloc” resection, curettage, and percutaneous drill excision of OO. The problem of open resection techniques is that the amount of tissue removed is often much larger than the tumor mass, mainly due to the difficulty of proper intraoperative nidus localization [3]. Percutaneous excision, on the other hand, is usually performed with relatively large-caliber needles and drills, and multiple drillings are sometimes necessary to ensure complete removal of the nidus [10].

Newer, less invasive percutaneous techniques, including chemical destruction and thermal ablation, have higher success rates and fewer complications. With thermal laser ablation (LA), for instance, the insertion of an 18-G needle is usually sufficient to destroy the entire nidus. Among the percutaneous modalities, CT-guided hyperthermal ablation using radiofrequency (RFA) or LA is currently the treatment of choice with success rates of more than 90% [7, 11, 12]. In the treatment of monocentric OO, LA and RFA have comparable outcomes in terms of primary success, recurrent pain, complications [8], and recurrence [2].

However, open MRI has features that may be advantageous for interventional guidance compared with CT, including superior soft tissue resolution and multiplanar navigation capabilities. The better resolution enables more reliable identification of critical tissue structures such as vessels, nerves, and cartilage in areas near the joints. Finally, the absence of ionizing radiation in MRI is advantageous especially in younger patients but also for the interventionist. 

MR-guided ablation is especially beneficial in the case of multicentric OO since the time needed for the intervention is longer than the time needed for eliminating a single nidus. On the other hand, MR guidance is more susceptible to instrument-induced artifacts, which may degrade the precision of the intervention. The occurrence of artifacts depends on the field strength used as well as the material the instruments are made of and their position relative to the main magnetic field B0. A major drawback of MRI guidance is that the size of the needle is usually overestimated due to artifact, which is most disturbing in critical regions such the periarticular region, the spine, and the small bones of the hands and feet [13], where CT usually enables more precise and safe needle placement.

Thermal monitoring of LA was reported to be unsuccessful in an open low-field scanner (0.23 T) [14]. However, successful thermal monitoring in an open 1.0 T MRI scanner has been described recently [9]. The laser effect was reliably identified by a signal void in the treated area (T1 method). The phase information and color-coded technique were found to be valuable in addition to conventional magnitude images (proton resonance frequency (PRF) method). Compared with RFA, the laser technique is fully MRI compatible. Hence, online MR thermometry without the need to discontinue thermal ablation is possible, which may render the procedure more controllable and safe.

As shown in the direct postinterventional control, subtraction images are useful to check whether ablation has been successful. Unfortunately, our patient refused the administration of contrast agent in the follow-up MRI, due to an extreme fear of needles, though the disappearance of femoral bone marrow edema was found to be consistent with the relief of symptoms.

No tissue was sampled for histological confirmation of the diagnosis since the clinical and imaging findings suggested the diagnosis of OO. However, even specimens from surgical resection enable histological confirmation of OO in only 57–79% of the cases [15].

A recent study has shown that MR-guided LA is less expensive than CT-guided RFA, mainly due to the higher costs of the disposable RF ablation probes used in CT-guided interventions [16].

To our knowledge, this report presents the first case of multicentric osteoid osteoma treated with laser ablation under open high-field MRI guidance. In the reported case, LA under open MRI guidance was safe and effective. The avoidance of ionizing radiation when MRI guidance is used is advantageous, especially when the patient is young and two nidi have to be removed, as in our case. Still, further studies are needed to determine whether the theoretical advantages of open MRI-guided laser ablation can also be confirmed in larger patient collectives.
